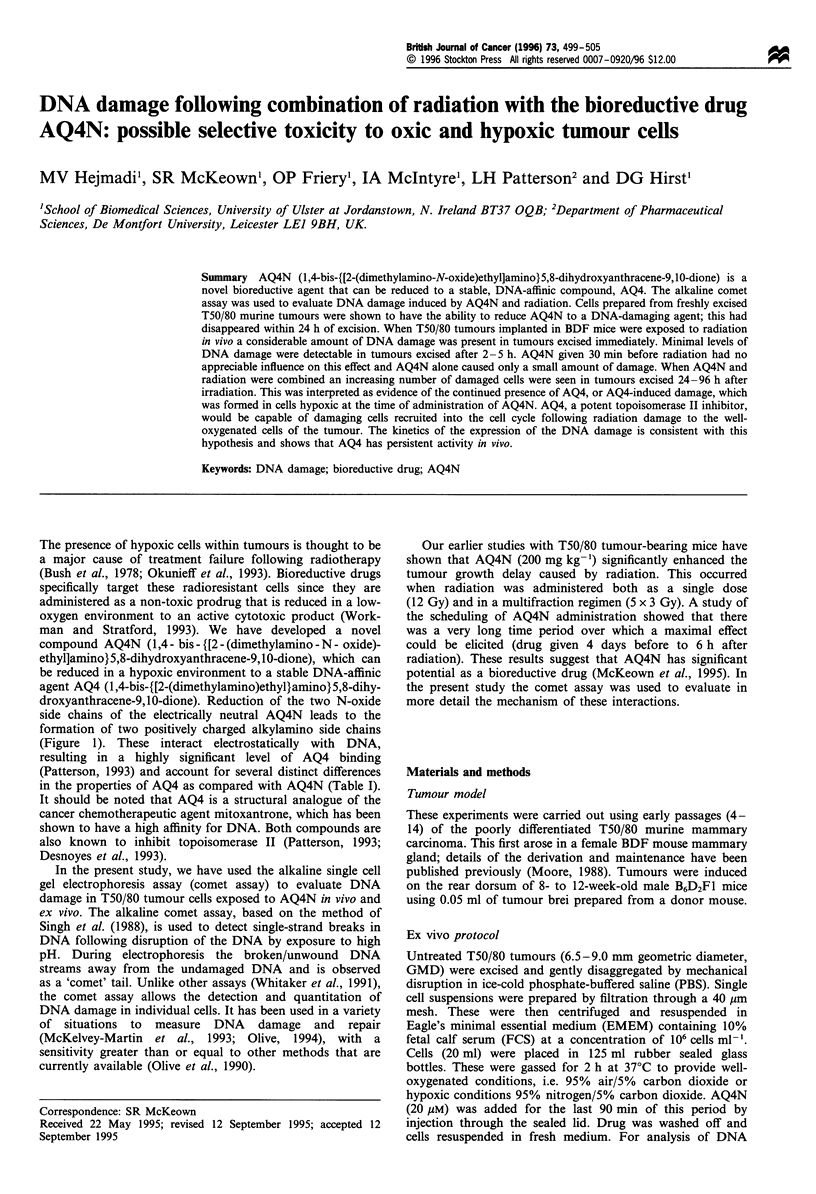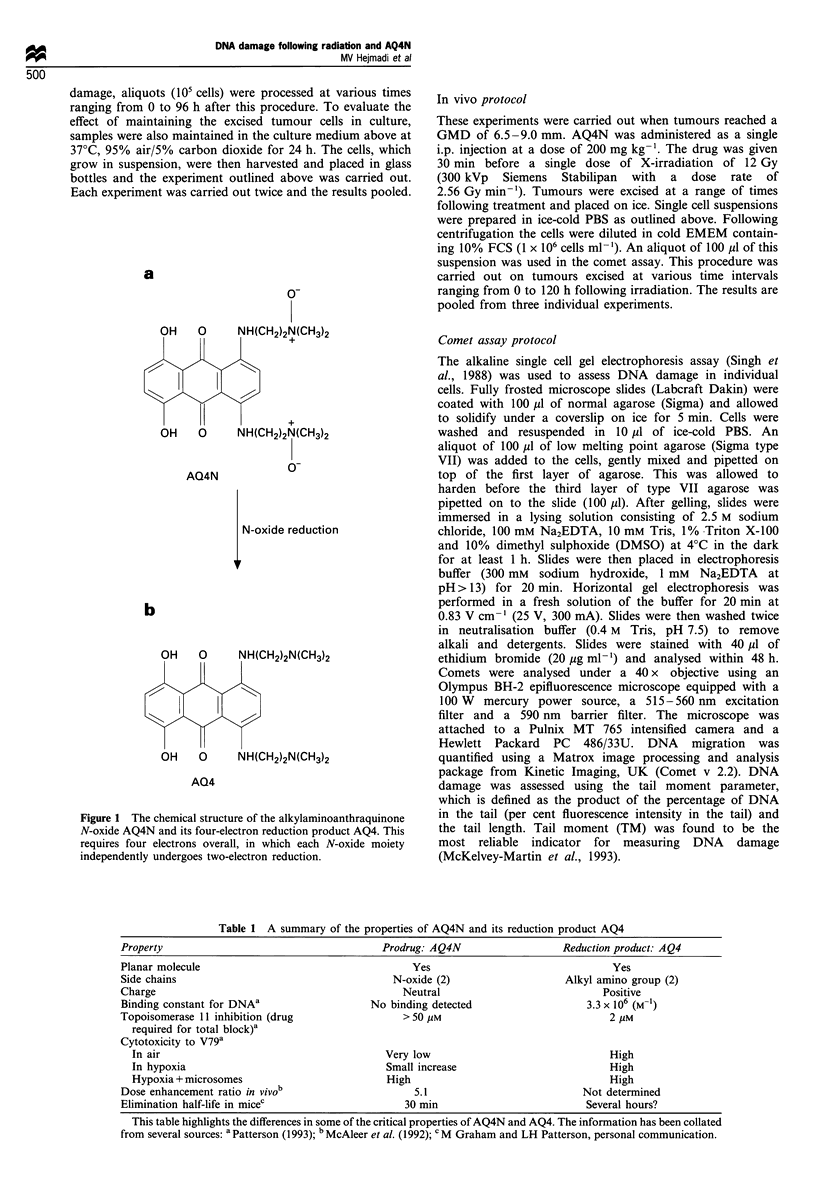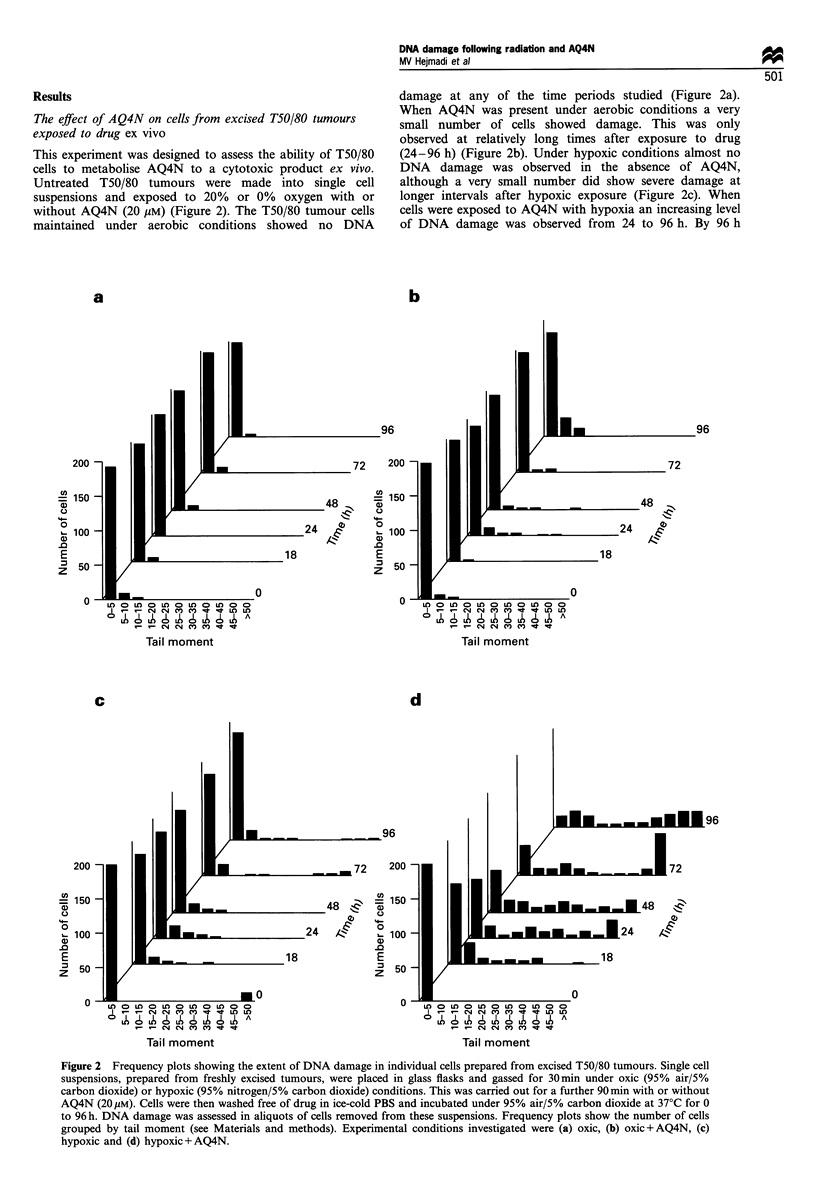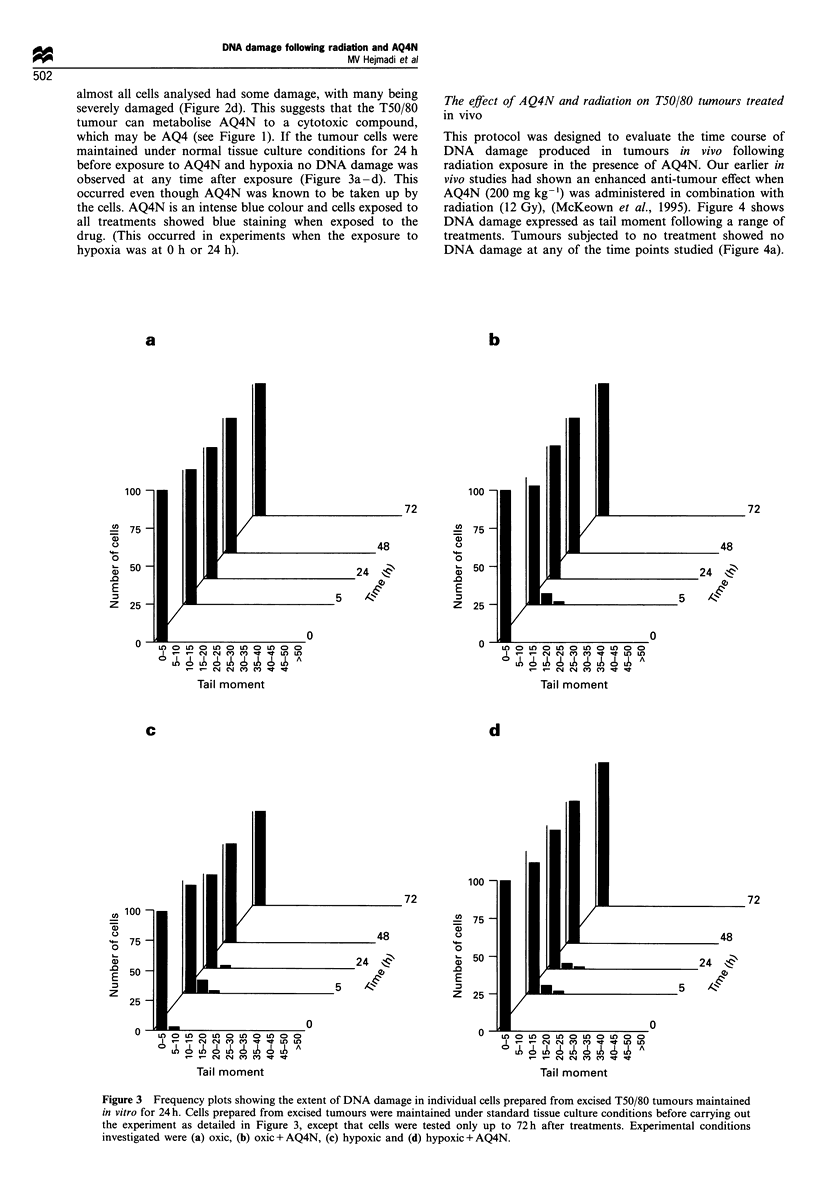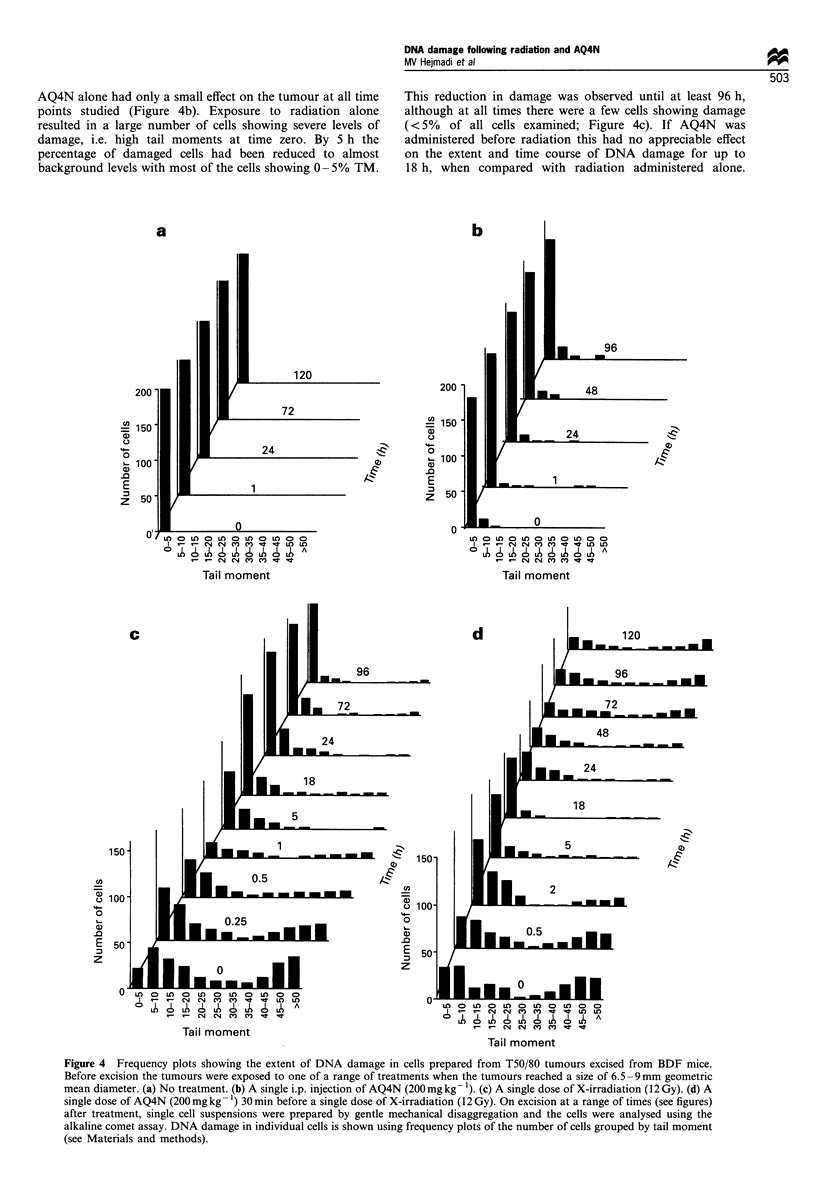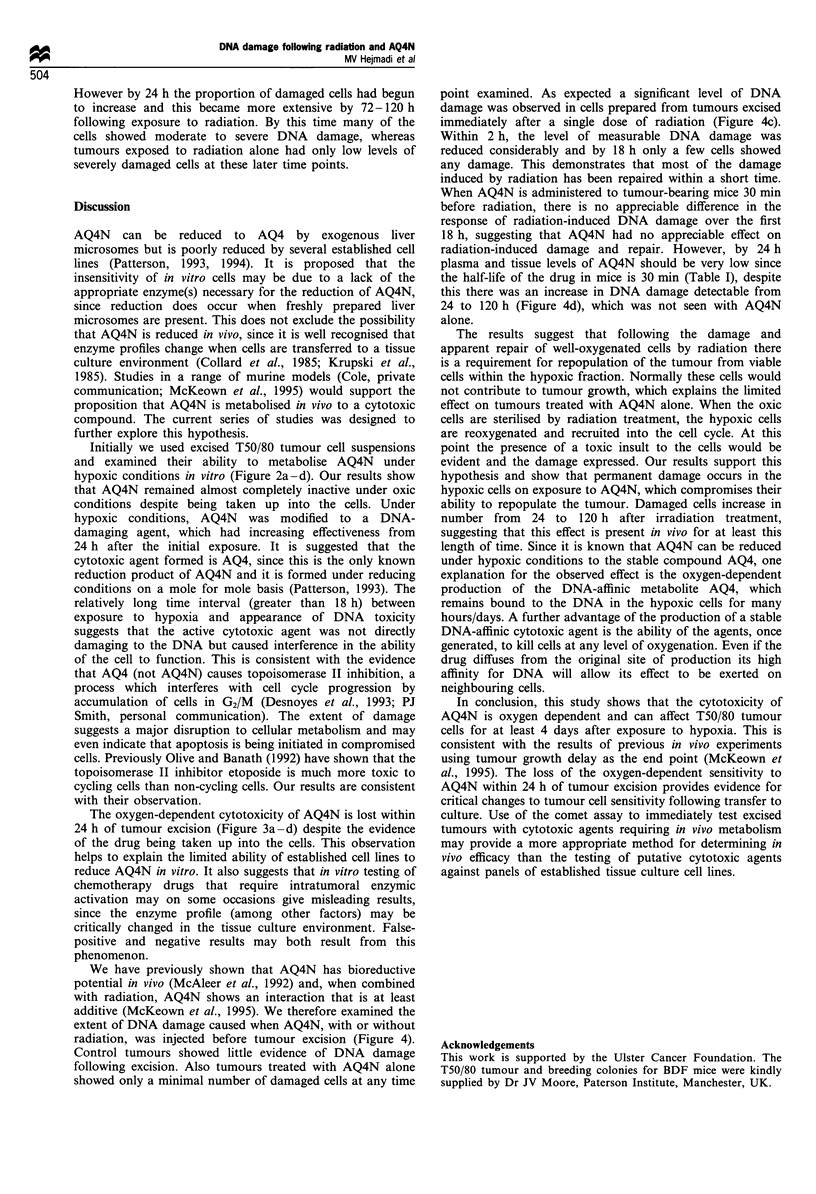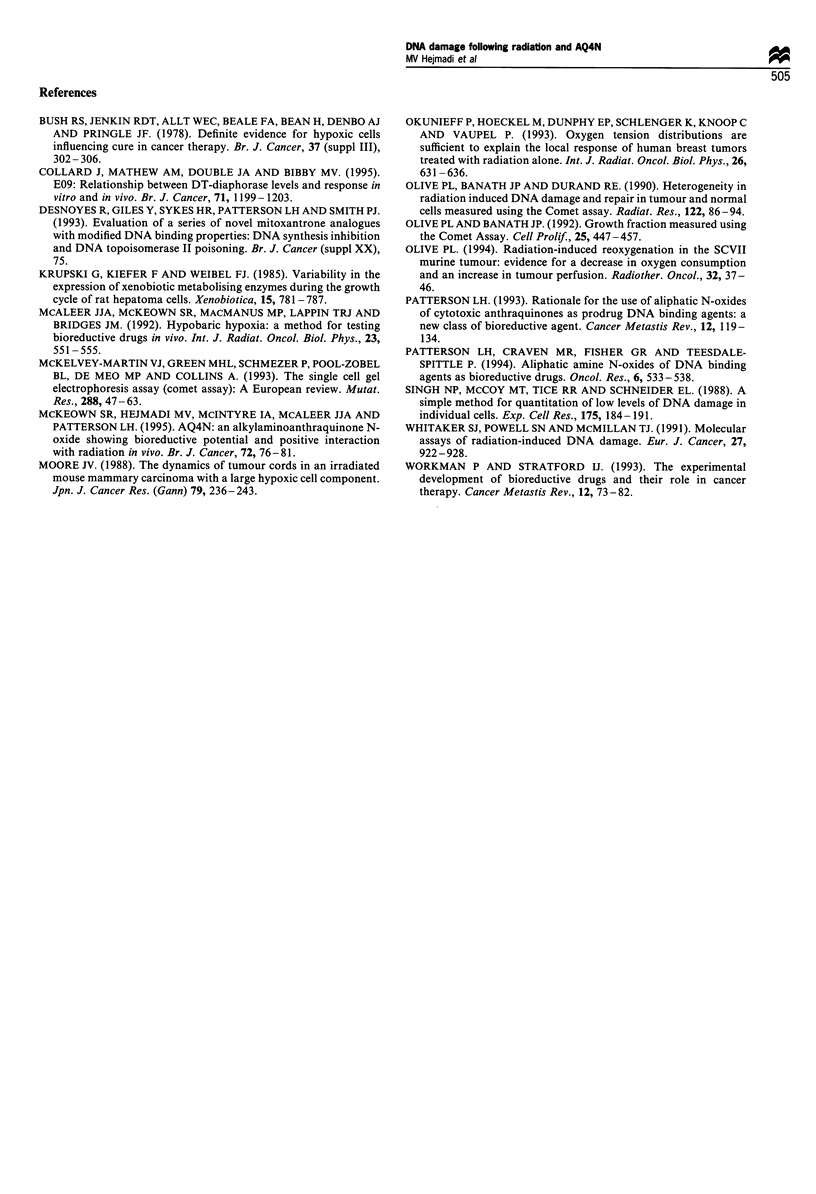# DNA damage following combination of radiation with the bioreductive drug AQ4N: possible selective toxicity to oxic and hypoxic tumour cells.

**DOI:** 10.1038/bjc.1996.87

**Published:** 1996-02

**Authors:** M. V. Hejmadi, S. R. McKeown, O. P. Friery, I. A. McIntyre, L. H. Patterson, D. G. Hirst

**Affiliations:** School of Biomedical Science, University of Ulster at Jordanstown, N. Ireland, UK.

## Abstract

AQ4N (1,4-bis-([2-(dimethylamino-N- oxide)ethyl]amino)5,8-dihydroxyanthracene-9,10-dione) is a novel bioreductive agent that can be reduced to a stable, DNA-affinic compound, AQ4. The alkaline comet assay was used to evaluate DNA damage induced by AQ4N and radiation. Cells prepared from freshly excised T50/80 murine tumours were shown to have the ability to reduce AQ4N to a DNA-damaging agent; this had disappeared within 24 h of excision. When T50/80 tumours implanted in BDF mice were exposed to radiation in vivo a considerable amount of DNA damage was present in tumours excised immediately. Minimal levels of DNA damage were detectable in tumours excised after 2-5 h. AQ4N given 30 min before radiation had no appreciable influence on this effect and AQ4N alone caused only a small amount of damage. When AQ4N and radiation were combined an increasing number of damaged cells were seen in tumours excised 24-96 h after irradiation. This was interpreted as evidence of the continued presence of AQ4, or AQ4-induced damage, which was formed in cells hypoxic at the time of administration of AQ4N. AQ4, a potent topoisomerase II inhibitor, would be capable of damaging cells recruited into the cell cycle following radiation damage to the well-oxygenated cells of the tumour. The kinetics of the expression of the DNA damage is consistent with this hypothesis and shows that AQ4 has persistent activity in vivo.


British Journal of Cancer (1996) 73, 499-505
© 1996 Stockton Press All rights reserved 0007-0920/96 $12.00

### DNA damage following combination of radiation with the bioreductive drug AQ4N: possible selective toxicity to oxic and hypoxic tumour cells

MV Hejmadi[1], SR McKeown[1], OP Friery[1], IA McIntyre[1], LH Patterson[2] and DG Hirst[1]

[1]*School of Biomedical Sciences, University of Ulster at Jordanstown, N. Ireland BT37 OQB;* [2]*Department of Pharmaceutical Sciences, De Montfort University, Leicester LE1 9BH, UK.*

**Summary** AQ4N (1,4-bis-{[2-(dimethylamino-*N*-oxide)ethyl]amino}5,8-dihydroxyanthracene-9,10-dione) is a novel bioreductive agent that can be reduced to a stable, DNA-affinic compound, AQ4. The alkaline comet assay was used to evaluate DNA damage induced by AQ4N and radiation. Cells prepared from freshly excised T50/80 murine tumours were shown to have the ability to reduce AQ4N to a DNA-damaging agent; this had disappeared within 24 h of excision. When T50/80 tumours implanted in BDF mice were exposed to radiation *in vivo* a considerable amount of DNA damage was present in tumours excised immediately. Minimal levels of DNA damage were detectable in tumours excised after 2-5 h. AQ4N given 30 min before radiation had no appreciable influence on this effect and AQ4N alone caused only a small amount of damage. When AQ4N and radiation were combined an increasing number of damaged cells were seen in tumours excised 24-96 h after irradiation. This was interpreted as evidence of the continued presence of AQ4, or AQ4-induced damage, which was formed in cells hypoxic at the time of administration of AQ4N. AQ4, a potent topoisomerase II inhibitor, would be capable of damaging cells recruited into the cell cycle following radiation damage to the well-oxygenated cells of the tumour. The kinetics of the expression of the DNA damage is consistent with this hypothesis and shows that AQ4 has persistent activity *in vivo*.

**Keywords:** DNA damage; bioreductive drug; AQ4N

The presence of hypoxic cells within tumours is thought to be a major cause of treatment failure following radiotherapy (Bush *et al.*, 1978; Okunieff *et al.*, 1993). Bioreductive drugs specifically target these radioresistant cells since they are administered as a non-toxic prodrug that is reduced in a low-oxygen environment to an active cytotoxic product (Workman and Stratford, 1993). We have developed a novel compound AQ4N (1,4- bis - {[2 - (dimethylamino - N - oxide)-ethyl]amino}5,8-dihydroxyanthracene-9,10-dione), which can be reduced in a hypoxic environment to a stable DNA-affinic agent AQ4 (1,4-bis-{[2-(dimethylamino)ethyl}amino}5,8-dihydroxyanthracene-9,10-dione). Reduction of the two N-oxide side chains of the electrically neutral AQ4N leads to the formation of two positively charged alkylamino side chains (Figure 1). These interact electrostatically with DNA, resulting in a highly significant level of AQ4 binding (Patterson, 1993) and account for several distinct differences in the properties of AQ4 as compared with AQ4N (Table I). It should be noted that AQ4 is a structural analogue of the cancer chemotherapeutic agent mitoxantrone, which has been shown to have a high affinity for DNA. Both compounds are also known to inhibit topoisomerase II (Patterson, 1993; Desnoyes *et al.*, 1993).

In the present study, we have used the alkaline single cell gel electrophoresis assay (comet assay) to evaluate DNA damage in T50/80 tumour cells exposed to AQ4N *in vivo* and *ex vivo*. The alkaline comet assay, based on the method of Singh *et al.* (1988), is used to detect single-strand breaks in DNA following disruption of the DNA by exposure to high pH. During electrophoresis the broken/unwound DNA streams away from the undamaged DNA and is observed as a 'comet' tail. Unlike other assays (Whitaker *et al.*, 1991), the comet assay allows the detection and quantitation of DNA damage in individual cells. It has been used in a variety of situations to measure DNA damage and repair (McKelvey-Martin *et al.*, 1993; Olive, 1994), with a sensitivity greater than or equal to other methods that are currently available (Olive *et al.*, 1990).

Correspondence: SR McKeown
Received 22 May 1995; revised 12 September 1995; accepted 12 September 1995

Our earlier studies with T50/80 tumour-bearing mice have shown that AQ4N (200 mg $kg^{-1}$) significantly enhanced the tumour growth delay caused by radiation. This occurred when radiation was administered both as a single dose (12 Gy) and in a multifraction regimen (5 × 3 Gy). A study of the scheduling of AQ4N administration showed that there was a very long time period over which a maximal effect could be elicited (drug given 4 days before to 6 h after radiation). These results suggest that AQ4N has significant potential as a bioreductive drug (McKeown *et al.*, 1995). In the present study the comet assay was used to evaluate in more detail the mechanism of these interactions.

### Materials and methods

### *Tumour model*

These experiments were carried out using early passages (4-14) of the poorly differentiated T50/80 murine mammary carcinoma. This first arose in a female BDF mouse mammary gland; details of the derivation and maintenance have been published previously (Moore, 1988). Tumours were induced on the rear dorsum of 8- to 12-week-old male $B_6D_2F1$ mice using 0.05 ml of tumour brei prepared from a donor mouse.

### Ex vivo *protocol*

Untreated T50/80 tumours (6.5-9.0 mm geometric diameter, GMD) were excised and gently disaggregated by mechanical disruption in ice-cold phosphate-buffered saline (PBS). Single cell suspensions were prepared by filtration through a 40 $\mu$m mesh. These were then centrifuged and resuspended in Eagle's minimal essential medium (EMEM) containing 10% fetal calf serum (FCS) at a concentration of $10^6$ cells $ml^{-1}$. Cells (20 ml) were placed in 125 ml rubber sealed glass bottles. These were gassed for 2 h at 37°C to provide well-oxygenated conditions, i.e. 95% air/5% carbon dioxide or hypoxic conditions 95% nitrogen/5% carbon dioxide. AQ4N (20 $\mu$M) was added for the last 90 min of this period by injection through the sealed lid. Drug was washed off and cells resuspended in fresh medium. For analysis of DNA

damage, aliquots ($10^5$ cells) were processed at various times ranging from 0 to 96 h after this procedure. To evaluate the effect of maintaining the excised tumour cells in culture, samples were also maintained in the culture medium above at 37°C, 95% air/5% carbon dioxide for 24 h. The cells, which grow in suspension, were then harvested and placed in glass bottles and the experiment outlined above was carried out. Each experiment was carried out twice and the results pooled.

**Figure 1** The chemical structure of the alkylaminoanthraquinone *N*-oxide AQ4N and its four-electron reduction product AQ4. This requires four electrons overall, in which each *N*-oxide moiety independently undergoes two-electron reduction.

*In vivo protocol*

These experiments were carried out when tumours reached a GMD of 6.5–9.0 mm. AQ4N was administered as a single i.p. injection at a dose of 200 mg $kg^{-1}$. The drug was given 30 min before a single dose of X-irradiation of 12 Gy (300 kVp Siemens Stabilipan with a dose rate of 2.56 Gy $min^{-1}$). Tumours were excised at a range of times following treatment and placed on ice. Single cell suspensions were prepared in ice-cold PBS as outlined above. Following centrifugation the cells were diluted in cold EMEM containing 10% FCS ($1 \times 10^6$ cells $ml^{-1}$). An aliquot of 100 $\mu$l of this suspension was used in the comet assay. This procedure was carried out on tumours excised at various time intervals ranging from 0 to 120 h following irradiation. The results are pooled from three individual experiments.

*Comet assay protocol*

The alkaline single cell gel electrophoresis assay (Singh *et al.*, 1988) was used to assess DNA damage in individual cells. Fully frosted microscope slides (Labcraft Dakin) were coated with 100 $\mu$l of normal agarose (Sigma) and allowed to solidify under a coverslip on ice for 5 min. Cells were washed and resuspended in 10 $\mu$l of ice-cold PBS. An aliquot of 100 $\mu$l of low melting point agarose (Sigma type VII) was added to the cells, gently mixed and pipetted on top of the first layer of agarose. This was allowed to harden before the third layer of type VII agarose was pipetted on to the slide (100 $\mu$l). After gelling, slides were immersed in a lysing solution consisting of 2.5 M sodium chloride, 100 mM $Na_2EDTA$, 10 mM Tris, 1% Triton X-100 and 10% dimethyl sulphoxide (DMSO) at 4°C in the dark for at least 1 h. Slides were then placed in electrophoresis buffer (300 mM sodium hydroxide, 1 mM $Na_2EDTA$ at pH > 13) for 20 min. Horizontal gel electrophoresis was performed in a fresh solution of the buffer for 20 min at 0.83 V $cm^{-1}$ (25 V, 300 mA). Slides were then washed twice in neutralisation buffer (0.4 M Tris, pH 7.5) to remove alkali and detergents. Slides were stained with 40 $\mu$l of ethidium bromide (20 $\mu$g $ml^{-1}$) and analysed within 48 h. Comets were analysed under a 40× objective using an Olympus BH-2 epifluorescence microscope equipped with a 100 W mercury power source, a 515–560 nm excitation filter and a 590 nm barrier filter. The microscope was attached to a Pulnix MT 765 intensified camera and a Hewlett Packard PC 486/33U. DNA migration was quantified using a Matrox image processing and analysis package from Kinetic Imaging, UK (Comet v 2.2). DNA damage was assessed using the tail moment parameter, which is defined as the product of the percentage of DNA in the tail (per cent fluorescence intensity in the tail) and the tail length. Tail moment (TM) was found to be the most reliable indicator for measuring DNA damage (McKelvey-Martin *et al.*, 1993).

**Table 1** A summary of the properties of AQ4N and its reduction product AQ4

| *Property* | *Prodrug: AQ4N* | *Reduction product: AQ4* |
|---|---|---|
| Planar molecule | Yes | Yes |
| Side chains | N-oxide (2) | Alkyl amino group (2) |
| Charge | Neutral | Positive |
| Binding constant for DNA[a] | No binding detected | $3.3 \times 10^6$ ($M^{-1}$) |
| Topoisomerase 11 inhibition (drug required for total block)[a] | > 50 $\mu$M | 2 $\mu$M |
| Cytotoxicity to V79[a] | | |
| In air | Very low | High |
| In hypoxia | Small increase | High |
| Hypoxia + microsomes | High | High |
| Dose enhancement ratio *in vivo*[b] | 5.1 | Not determined |
| Elimination half-life in mice[c] | 30 min | Several hours? |

This table highlights the differences in some of the critical properties of AQ4N and AQ4. The information has been collated from several sources: [a] Patterson (1993); [b] McAleer *et al.* (1992); [c] M Graham and LH Patterson, personal communication.

### Results

*The effect of AQ4N on cells from excised T50/80 tumours exposed to drug* ex vivo

This experiment was designed to assess the ability of T50/80 cells to metabolise AQ4N to a cytotoxic product *ex vivo*. Untreated T50/80 tumours were made into single cell suspensions and exposed to 20% or 0% oxygen with or without AQ4N (20 $\mu$M) (Figure 2). The T50/80 tumour cells maintained under aerobic conditions showed no DNA damage at any of the time periods studied (Figure 2a). When AQ4N was present under aerobic conditions a very small number of cells showed damage. This was only observed at relatively long times after exposure to drug (24–96 h) (Figure 2b). Under hypoxic conditions almost no DNA damage was observed in the absence of AQ4N, although a very small number did show severe damage at longer intervals after hypoxic exposure (Figure 2c). When cells were exposed to AQ4N with hypoxia an increasing level of DNA damage was observed from 24 to 96 h. By 96 h

**Figure 2** Frequency plots showing the extent of DNA damage in individual cells prepared from excised T50/80 tumours. Single cell suspensions, prepared from freshly excised tumours, were placed in glass flasks and gassed for 30 min under oxic (95% air/5% carbon dioxide) or hypoxic (95% nitrogen/5% carbon dioxide) conditions. This was carried out for a further 90 min with or without AQ4N (20 $\mu$M). Cells were then washed free of drug in ice-cold PBS and incubated under 95% air/5% carbon dioxide at 37°C for 0 to 96 h. DNA damage was assessed in aliquots of cells removed from these suspensions. Frequency plots show the number of cells grouped by tail moment (see Materials and methods). Experimental conditions investigated were (**a**) oxic, (**b**) oxic + AQ4N, (**c**) hypoxic and (**d**) hypoxic + AQ4N.

almost all cells analysed had some damage, with many being severely damaged (Figure 2d). This suggests that the T50/80 tumour can metabolise AQ4N to a cytotoxic compound, which may be AQ4 (see Figure 1). If the tumour cells were maintained under normal tissue culture conditions for 24 h before exposure to AQ4N and hypoxia no DNA damage was observed at any time after exposure (Figure 3a–d). This occurred even though AQ4N was known to be taken up by the cells. AQ4N is an intense blue colour and cells exposed to all treatments showed blue staining when exposed to the drug. (This occurred in experiments when the exposure to hypoxia was at 0 h or 24 h).

*The effect of AQ4N and radiation on T50/80 tumours treated* in vivo

This protocol was designed to evaluate the time course of DNA damage produced in tumours *in vivo* following radiation exposure in the presence of AQ4N. Our earlier *in vivo* studies had shown an enhanced anti-tumour effect when AQ4N (200 mg $kg^{-1}$) was administered in combination with radiation (12 Gy), (McKeown *et al.*, 1995). Figure 4 shows DNA damage expressed as tail moment following a range of treatments. Tumours subjected to no treatment showed no DNA damage at any of the time points studied (Figure 4a).

**Figure 3** Frequency plots showing the extent of DNA damage in individual cells prepared from excised T50/80 tumours maintained *in vitro* for 24 h. Cells prepared from excised tumours were maintained under standard tissue culture conditions before carrying out the experiment as detailed in Figure 3, except that cells were tested only up to 72 h after treatments. Experimental conditions investigated were (**a**) oxic, (**b**) oxic + AQ4N, (**c**) hypoxic and (**d**) hypoxic + AQ4N.

AQ4N alone had only a small effect on the tumour at all time points studied (Figure 4b). Exposure to radiation alone resulted in a large number of cells showing severe levels of damage, i.e. high tail moments at time zero. By 5 h the percentage of damaged cells had been reduced to almost background levels with most of the cells showing 0–5% TM. This reduction in damage was observed until at least 96 h, although at all times there were a few cells showing damage (<5% of all cells examined; Figure 4c). If AQ4N was administered before radiation this had no appreciable effect on the extent and time course of DNA damage for up to 18 h, when compared with radiation administered alone.

**Figure 4** Frequency plots showing the extent of DNA damage in cells prepared from T50/80 tumours excised from BDF mice. Before excision the tumours were exposed to one of a range of treatments when the tumours reached a size of 6.5–9 mm geometric mean diameter. (**a**) No treatment. (**b**) A single i.p. injection of AQ4N (200 mg kg$^{-1}$). (**c**) A single dose of X-irradiation (12 Gy). (**d**) A single dose of AQ4N (200 mg kg$^{-1}$) 30 min before a single dose of X-irradiation (12 Gy). On excision at a range of times (see figures) after treatment, single cell suspensions were prepared by gentle mechanical disaggregation and the cells were analysed using the alkaline comet assay. DNA damage in individual cells is shown using frequency plots of the number of cells grouped by tail moment (see Materials and methods).

However by 24 h the proportion of damaged cells had begun to increase and this became more extensive by 72–120 h following exposure to radiation. By this time many of the cells showed moderate to severe DNA damage, whereas tumours exposed to radiation alone had only low levels of severely damaged cells at these later time points.

### Discussion

AQ4N can be reduced to AQ4 by exogenous liver microsomes but is poorly reduced by several established cell lines (Patterson, 1993, 1994). It is proposed that the insensitivity of *in vitro* cells may be due to a lack of the appropriate enzyme(s) necessary for the reduction of AQ4N, since reduction does occur when freshly prepared liver microsomes are present. This does not exclude the possibility that AQ4N is reduced *in vivo*, since it is well recognised that enzyme profiles change when cells are transferred to a tissue culture environment (Collard *et al.*, 1985; Krupski *et al.*, 1985). Studies in a range of murine models (Cole, private communication; McKeown *et al.*, 1995) would support the proposition that AQ4N is metabolised *in vivo* to a cytotoxic compound. The current series of studies was designed to further explore this hypothesis.

Initially we used excised T50/80 tumour cell suspensions and examined their ability to metabolise AQ4N under hypoxic conditions *in vitro* (Figure 2a–d). Our results show that AQ4N remained almost completely inactive under oxic conditions despite being taken up into the cells. Under hypoxic conditions, AQ4N was modified to a DNA-damaging agent, which had increasing effectiveness from 24 h after the initial exposure. It is suggested that the cytotoxic agent formed is AQ4, since this is the only known reduction product of AQ4N and it is formed under reducing conditions on a mole for mole basis (Patterson, 1993). The relatively long time interval (greater than 18 h) between exposure to hypoxia and appearance of DNA toxicity suggests that the active cytotoxic agent was not directly damaging to the DNA but caused interference in the ability of the cell to function. This is consistent with the evidence that AQ4 (not AQ4N) causes topoisomerase II inhibition, a process which interferes with cell cycle progression by accumulation of cells in $G_2$/M (Desnoyes *et al.*, 1993; PJ Smith, personal communication). The extent of damage suggests a major disruption to cellular metabolism and may even indicate that apoptosis is being initiated in compromised cells. Previously Olive and Banath (1992) have shown that the topoisomerase II inhibitor etoposide is much more toxic to cycling cells than non-cycling cells. Our results are consistent with their observation.

The oxygen-dependent cytotoxicity of AQ4N is lost within 24 h of tumour excision (Figure 3a–d) despite the evidence of the drug being taken up into the cells. This observation helps to explain the limited ability of established cell lines to reduce AQ4N *in vitro*. It also suggests that *in vitro* testing of chemotherapy drugs that require intratumoral enzymic activation may on some occasions give misleading results, since the enzyme profile (among other factors) may be critically changed in the tissue culture environment. False-positive and negative results may both result from this phenomenon.

We have previously shown that AQ4N has bioreductive potential *in vivo* (McAleer *et al.*, 1992) and, when combined with radiation, AQ4N shows an interaction that is at least additive (McKeown *et al.*, 1995). We therefore examined the extent of DNA damage caused when AQ4N, with or without radiation, was injected before tumour excision (Figure 4). Control tumours showed little evidence of DNA damage following excision. Also tumours treated with AQ4N alone showed only a minimal number of damaged cells at any time point examined. As expected a significant level of DNA damage was observed in cells prepared from tumours excised immediately after a single dose of radiation (Figure 4c). Within 2 h, the level of measurable DNA damage was reduced considerably and by 18 h only a few cells showed any damage. This demonstrates that most of the damage induced by radiation has been repaired within a short time. When AQ4N is administered to tumour-bearing mice 30 min before radiation, there is no appreciable difference in the response of radiation-induced DNA damage over the first 18 h, suggesting that AQ4N had no appreciable effect on radiation-induced damage and repair. However, by 24 h plasma and tissue levels of AQ4N should be very low since the half-life of the drug in mice is 30 min (Table I), despite this there was an increase in DNA damage detectable from 24 to 120 h (Figure 4d), which was not seen with AQ4N alone.

The results suggest that following the damage and apparent repair of well-oxygenated cells by radiation there is a requirement for repopulation of the tumour from viable cells within the hypoxic fraction. Normally these cells would not contribute to tumour growth, which explains the limited effect on tumours treated with AQ4N alone. When the oxic cells are sterilised by radiation treatment, the hypoxic cells are reoxygenated and recruited into the cell cycle. At this point the presence of a toxic insult to the cells would be evident and the damage expressed. Our results support this hypothesis and show that permanent damage occurs in the hypoxic cells on exposure to AQ4N, which compromises their ability to repopulate the tumour. Damaged cells increase in number from 24 to 120 h after irradiation treatment, suggesting that this effect is present *in vivo* for at least this length of time. Since it is known that AQ4N can be reduced under hypoxic conditions to the stable compound AQ4, one explanation for the observed effect is the oxygen-dependent production of the DNA-affinic metabolite AQ4, which remains bound to the DNA in the hypoxic cells for many hours/days. A further advantage of the production of a stable DNA-affinic cytotoxic agent is the ability of the agents, once generated, to kill cells at any level of oxygenation. Even if the drug diffuses from the original site of production its high affinity for DNA will allow its effect to be exerted on neighbouring cells.

In conclusion, this study shows that the cytotoxicity of AQ4N is oxygen dependent and can affect T50/80 tumour cells for at least 4 days after exposure to hypoxia. This is consistent with the results of previous *in vivo* experiments using tumour growth delay as the end point (McKeown *et al.*, 1995). The loss of the oxygen-dependent sensitivity to AQ4N within 24 h of tumour excision provides evidence for critical changes to tumour cell sensitivity following transfer to culture. Use of the comet assay to immediately test excised tumours with cytotoxic agents requiring *in vivo* metabolism may provide a more appropriate method for determining *in vivo* efficacy than the testing of putative cytotoxic agents against panels of established tissue culture cell lines.

**Acknowledgements**

This work is supported by the Ulster Cancer Foundation. The T50/80 tumour and breeding colonies for BDF mice were kindly supplied by Dr JV Moore, Paterson Institute, Manchester, UK.

### References

BUSH RS, JENKIN RDT, ALLT WEC, BEALE FA, BEAN H, DENBO AJ AND PRINGLE JF. (1978). Definite evidence for hypoxic cells influencing cure in cancer therapy. *Br. J. Cancer*, **37** (suppl III), 302–306.

COLLARD J, MATHEW AM, DOUBLE JA AND BIBBY MV. (1995). E09: Relationship between DT-diaphorase levels and response *in vitro* and *in vivo*. *Br. J. Cancer*, **71**, 1199–1203.

DESNOYES R, GILES Y, SYKES HR, PATTERSON LH AND SMITH PJ. (1993). Evaluation of a series of novel mitoxantrone analogues with modified DNA binding properties: DNA synthesis inhibition and DNA topoisomerase II poisoning. *Br. J. Cancer* (suppl XX), 75.

KRUPSKI G, KIEFER F AND WEIBEL FJ. (1985). Variability in the expression of xenobiotic metabolising enzymes during the growth cycle of rat hepatoma cells. *Xenobiotica*, **15**, 781–787.

MCALEER JJA, MCKEOWN SR, MACMANUS MP, LAPPIN TRJ AND BRIDGES JM. (1992). Hypobaric hypoxia: a method for testing bioreductive drugs *in vivo*. *Int. J. Radiat. Oncol. Biol. Phys.*, **23**, 551–555.

MCKELVEY-MARTIN VJ, GREEN MHL, SCHMEZER P, POOL-ZOBEL BL, DE MEO MP AND COLLINS A. (1993). The single cell gel electrophoresis assay (comet assay): A European review. *Mutat. Res.*, **288**, 47–63.

MCKEOWN SR, HEJMADI MV, MCINTYRE IA, MCALEER JJA AND PATTERSON LH. (1995). AQ4N: an alkylaminoanthraquinone N-oxide showing bioreductive potential and positive interaction with radiation *in vivo*. *Br. J. Cancer*, **72**, 76–81.

MOORE JV. (1988). The dynamics of tumour cords in an irradiated mouse mammary carcinoma with a large hypoxic cell component. *Jpn. J. Cancer Res.* (*Gann*) **79**, 236–243.

OKUNIEFF P, HOECKEL M, DUNPHY EP, SCHLENGER K, KNOOP C AND VAUPEL P. (1993). Oxygen tension distributions are sufficient to explain the local response of human breast tumors treated with radiation alone. *Int. J. Radiat. Oncol. Biol. Phys.*, **26**, 631–636.

OLIVE PL, BANATH JP AND DURAND RE. (1990). Heterogeneity in radiation induced DNA damage and repair in tumour and normal cells measured using the Comet assay. *Radiat. Res.*, **122**, 86–94.

OLIVE PL AND BANATH JP. (1992). Growth fraction measured using the Comet Assay. *Cell Prolif.*, **25**, 447–457.

OLIVE PL. (1994). Radiation-induced reoxygenation in the SCVII murine tumour: evidence for a decrease in oxygen consumption and an increase in tumour perfusion. *Radiother. Oncol.*, **32**, 37–46.

PATTERSON LH. (1993). Rationale for the use of aliphatic N-oxides of cytotoxic anthraquinones as prodrug DNA binding agents: a new class of bioreductive agent. *Cancer Metastis Rev.*, **12**, 119–134.

PATTERSON LH, CRAVEN MR, FISHER GR AND TEESDALE-SPITTLE P. (1994). Aliphatic amine N-oxides of DNA binding agents as bioreductive drugs. *Oncol. Res.*, **6**, 533–538.

SINGH NP, MCCOY MT, TICE RR AND SCHNEIDER EL. (1988). A simple method for quantitation of low levels of DNA damage in individual cells. *Exp. Cell Res.*, **175**, 184–191.

WHITAKER SJ, POWELL SN AND MCMILLAN TJ. (1991). Molecular assays of radiation-induced DNA damage. *Eur. J. Cancer*, **27**, 922–928.

WORKMAN P AND STRATFORD IJ. (1993). The experimental development of bioreductive drugs and their role in cancer therapy. *Cancer Metastis Rev.*, **12**, 73–82.